# Smy2p Participates in COPII Vesicle Formation Through the Interaction with Sec23p/Sec24p Subcomplex

**DOI:** 10.1111/j.1600-0854.2007.00668.x

**Published:** 2007-11-27

**Authors:** Hironori Higashio, Ken Sato, Akihiko Nakano

**Affiliations:** 1 Molecular Membrane Biology Laboratory, RIKEN Discovery Research Institute 2-1 Hirosawa, Wako, Saitama 351-0198, Japan; 4 Department of Biological Sciences, Graduate School of Science, University of Tokyo 7-3-1 Hongo, Bunkyo-ku, Tokyo 113-0033, Japan

**Keywords:** coat protein complex II, endoplasmic reticulum, multicopy suppressor, *Saccharomyces cerevisiae*, *SEC24*, vesicle formation

## Abstract

The coat protein complex II (COPII) is essential for vesicle formation from the endoplasmic reticulum (ER) and is composed of two heterodimeric subcomplexes, Sec23p/Sec24p and Sec13p/Sec31p, and the small guanosine triphosphatase Sar1p. In an effort to identify novel factors that may participate in COPII vesicle formation, we isolated *SMY2*, a yeast gene encoding a protein of unknown function, as a multicopy suppressor of the temperature-sensitive *sec24-20* mutant. We found that even a low-copy expression of *SMY2* was sufficient for the suppression of the *sec24-20* phenotypes, and the chromosomal deletion of *SMY2* led to a severe growth defect in the *sec24-20* background. In addition, *SMY2* exhibited genetic interactions with several other genes involved in the ER-to-Golgi transport. Subcellular fractionation analysis showed that Smy2p was a peripheral membrane protein fractionating together with COPII components. However, Smy2p was not loaded onto COPII vesicles generated *in vitro*. Interestingly, coimmunoprecipitation between Smy2p and the Sec23p/Sec24p subcomplex was specifically observed in *sec23-1* and *sec24-20* backgrounds, suggesting that this interaction was a prerequisite for the suppression of the *sec24-20* phenotypes by overexpression of *SMY2*. We propose that Smy2p is located on the surface of the ER and facilitates COPII vesicle formation through the interaction with Sec23p/Sec24p subcomplex.

In eukaryotic cells, protein transport along the secretory pathway is mediated by transport vesicles, which emerge from a donor organelle and fuse with an appropriate target organelle. Newly synthesized secretory proteins are correctly folded in the endoplasmic reticulum (ER), which is the starting point of the secretory pathway, and then exported to the Golgi apparatus by coat protein complex II (COPII)-coated vesicles (1). COPII coat is essential for vesicle formation from the ER membrane and is composed of the small guanosine triphosphatase (GTPase) Sar1p (2) and heterodimeric subcomplexes, Sec23p/Sec24p and Sec13p/Sec31p (3). The vesicle formation starts with the conversion of inactive Sar1p-GDP to active Sar1p-GTP by Sec12p, the guanine-nucleotide exchange factor (GEF) for Sar1p, localized on the ER membrane (4, 5). Sar1p-GTP binds to the ER membrane and then sequentially recruits Sec23p/Sec24p and Sec13p/Sec31p to form a bud that is finally pinched off as a COPII vesicle (3). After formation, COPII vesicles lose their coats, and the resulting naked vesicles undergo tethering, docking and fusion to the Golgi membrane (6). Coat disassembly requires GTP hydrolysis of Sar1p, which is stimulated by Sec23p, the GTPase activating protein (GAP) for Sar1p (7, 8).

In addition to COPII vesicle formation, packaging of cargo molecules into vesicles is driven by the Sar1p–Sec23p/Sec24p prebudding complexes (9–11). Recent studies demonstrate that Sec24p contains several cargo-binding sites on its membrane-proximal surface and acts as the coat for cargo selection (12–14). The Sec24p ‘A-site’ recognizes YxxxNPF motif on the soluble *n*-ethylmaleimide-sensitive fusion protein attachment protein receptor (SNARE), Sed5p, and the ‘B-site’ binds multiple motifs: Lxx(L/M)E on the SNAREs, Sed5p and Bet1p, and (D/E)x(D/E) on the Golgi protein Sys1p (13, 14). The ‘C-site’ binds the SNARE Sec22p by recognizing its conformational epitope (13–15). Moreover, the existence of Sec24p subtypes in both yeast and mammals expands the cargo multiplicity captured by COPII coat (16–21). For example, *Saccharomyces cerevisiae* has two nonessential Sec24p homologues, Sfb2p (Iss1p) (56% identity) and Sfb3p (Lst1p) (23% identity), both of which can form a complex with Sec23p (16–19). Sfb3p is specialized for efficient packaging of the plasma membrane proton adenosine triphosphatase Pma1p into COPII vesicles (22), and chromosomal disruption of *SFB3* results in the pH-sensitive growth defect because of the arrest of Pma1p in the ER (19). In contrast, Sfb2p appears to be a functionally redundant Sec24p homologue because its overproduction not only suppresses the temperature-sensitive phenotypes of the *sec24* mutants but also replaces the essential gene *SEC24*(16–18).

Reconstitution studies have demonstrated that COPII components themselves are minimal requirements to drive vesicle formation from chemically defined liposomes (23–25). Although dispensable for the reconstitution, there are additional proteins involved in COPII vesicle formation *in vivo*: Sec16p, Sed4p and the Yip1p–Yif1p–Yos1p complex. Sec16p is a large, hydrophilic protein that associates peripherally with the ER membrane and can bind with Sec23p, Sec24p and Sec31p through its distinct domains (26–28). Sec16p is shown to bind to liposomes and facilitates the recruitment of COPII components and the following vesicle formation without regulating Sar1p-GTP hydrolysis (29), indicating that Sec16p is a scaffold for the assembly of COPII coats. In yeast *Pichia pastoris* and mammalian cells, Sec16p localizes to discrete subdomains of the ER termed transitional ER or ER exit sites and is involved in their organization as well as COPII vesicle formation (30, 31). Sed4p is an integral membrane protein localized on the ER, whose cytoplasmic domain shares 45% identity with that of Sec12p (32). *SED4* exhibits genetic interactions with *SEC12*, *SEC16* and *SAR1*(33, 34), and chromosomal disruption of *SED4* results in a decreased rate of ER-to-Golgi transport (33), suggesting the involvement of Sed4p in COPII vesicle formation. Finally, Yip1p is an integral membrane protein previously found to interact with several Rab GTPases including Ypt1p (35, 36) and forms a heterodimeric complex with its homologue Yif1p (37). From the observations that *YIP1* exhibits genetic interactions with *SEC12*, *SEC13* and *SEC23* and that ER membranes from the temperature-sensitive *yip1* allele show reduced COPII vesicle formation *in vitro*, Yip1p is likely implicated in COPII vesicle formation (38). Moreover, the Yip1p–Yif1p complex forms a ternary complex with another integral membrane protein Yos1p, whose defect also shows reduced COPII vesicle formation *in vitro*(39). Like Sec16p, Yip1A, a mammalian homologue of Yip1p, localizes to ER exit sites (40). Thus, it seems likely that the process by which COPII vesicles are formed is more complex *in vivo* than *in vitro*, and these observations prompt us to investigate other unknown proteins involved in COPII vesicle formation.

In this report, we characterized *SMY2*, a high-copy suppressor of the temperature-sensitive *sec24-20* mutant. While Sec24-20p can form a complex with Sec23p, the *sec24-20* mutation results in the severe blockage in ER-to-Golgi transport and the significant accumulation of the ER membrane without obvious vesicle accumulation at the restrictive temperature, strongly suggesting the defects in COPII vesicle formation (17). Our genetic and biochemical evidence suggests the involvement of Smy2p in COPII vesicle formation.

## Results

### *SMY2* is a novel suppressor of the *sec24-20* mutant

We screened a YEp13 (*LEU2*, *2μ*)-based yeast genomic DNA library (41) for high-copy suppressors of the temperature-sensitive *sec24-20* mutant and repeatedly obtained plasmids that harbored a genomic fragment containing six complete open reading frames (ORFs), YBR171w to YBR176w. After subcloning, YBR172c was found to be responsible for the suppression. This ORF remained to be characterized; however, it had already been named *SMY2* (*s*uppressor of *my*osin 2) because of its previous identification as a high-copy suppressor of the temperature-sensitive myosin V mutant *myo2-66*(42). Thus, we hereafter call this ORF *SMY2*.

**Figure 1:**
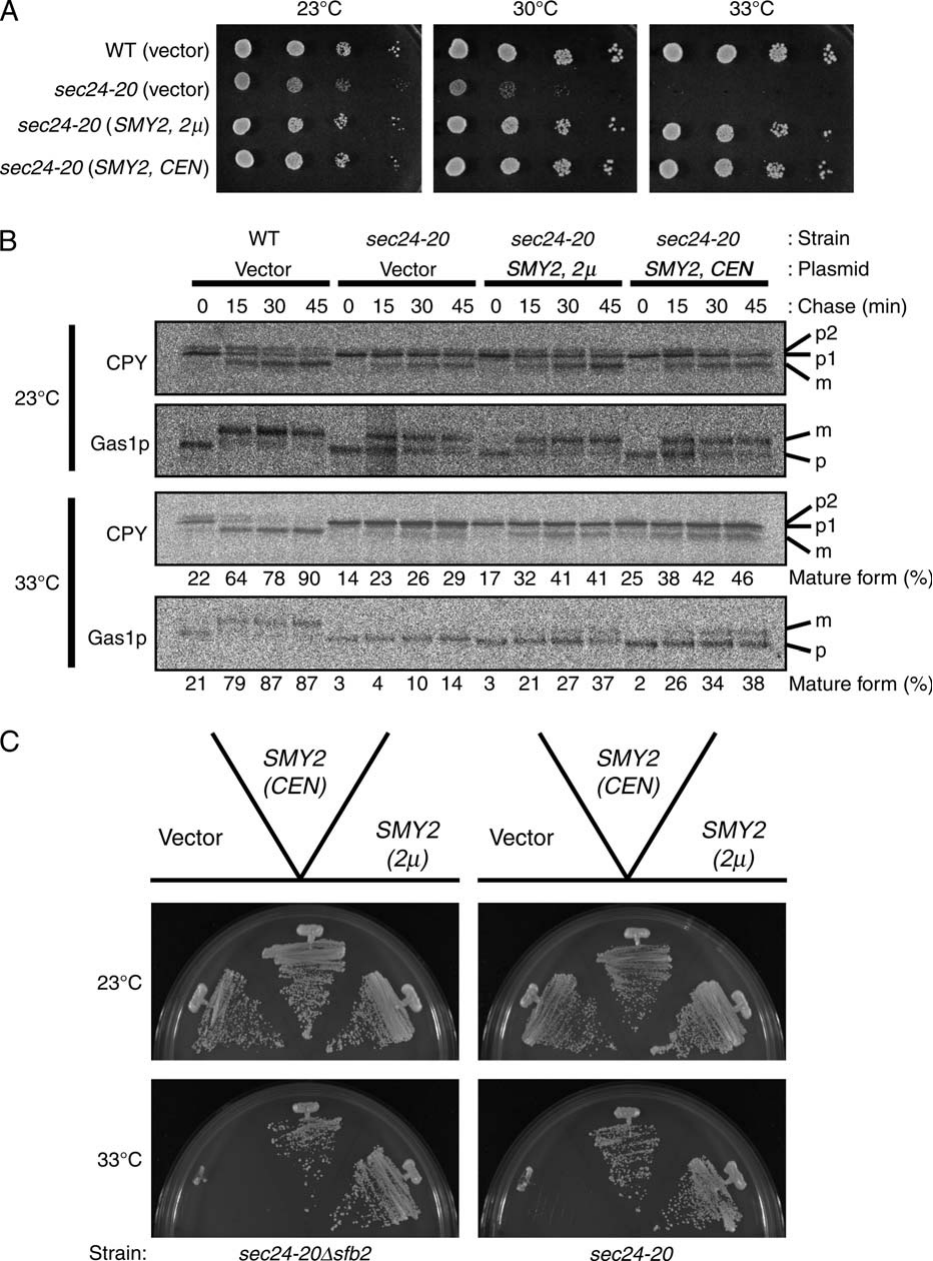
Suppression of *sec24-20* phenotypes by overexpression of *SMY2*. A) Wild-type (WT; YNH1) and *sec24-20* (YNH2) cells were transformed with pSMY1 (*SMY2*, *2μ*), pSMY3 (*SMY2*, *CEN*) or pRS426 (vector). Tenfold serial dilutions (starting from A_600_ of 0.1) of the transformants were spotted on MVD plates and incubated at the indicated temperatures for 3 days. B) The transformants used in A were incubated at 23°C or 33°C for 20 min, pulse labeled with [^35^S]-methionine/cysteine for 10 min and chased for the indicated times. After preparing cell lysates, CPY and Gas1p were immunoprecipitated, resolved by SDS–PAGE and visualized with a BAS2500 image analyzer. The different maturation forms of CPY (p1, ER form; p2, Golgi form; m, mature form in the vacuole) and Gas1p (p, ER form; m, mature form beyond the Golgi) and relative amounts of those mature forms at 33°C are indicated. C) *sec24-20* (YNH7) and *sec24-20Δsfb2* (YNH8) cells were transformed with pSMY1 (*SMY2*, *2μ*), pSMY3 (*SMY2*, *CEN*) or pRS426 (vector). The transformants were streaked onto MVD plates and incubated at the indicated temperatures for 3 days.

As shown in Figure 1A, the high-copy (*2μ*) and low-copy (*CEN*) plasmids containing *SMY2* suppressed the growth defect of *sec24-20* cells to the same extent at the restrictive temperature of 33°C. To directly observe whether the overexpression of *SMY2* rescues the ER-to-Golgi transport defect of *sec24-20* cells, we performed pulse–chase analysis of a vacuolar protein carboxypeptidase Y (CPY) and a glycosylphosphatidylinositol-anchored plasma membrane protein Gas1p (Figure 1B). Newly synthesized CPY is detected as the p1 precursor form (67 kDa) in the ER, further modified to the p2 form (69 kDa) in the Golgi and then proteolytically processed to the mature form (m; 61 kDa) in the vacuole (43). Similarly, Gas1p is detected as the 105-kDa precursor form (p) in the ER, further modified to the 125-kDa mature form (m) in the Golgi and then delivered to the plasma membrane (44, 45). As shown in Figure 1B, *sec24-20* cells exhibited severe maturation defects of both proteins and accumulated their ER forms (p1 of CPY and p of Gas1p) at the restrictive temperature of 33°C. These defects were partially suppressed by either high-copy (*2μ*) or low-copy (*CEN*) *SMY2* plasmid. These results indicate that even the low-copy (*CEN*) expression of *SMY2* is sufficient for the suppression of temperature-sensitive *sec24-20* phenotypes. As far as we examined, the overexpression of *SMY2* affected neither growth nor ER-to-Golgi transport in wild-type cells (data not shown).

We previously showed that overexpression of *SFB2* (*ISS1*) suppresses the *sec24-20* phenotypes (17). To address the relationship between *SMY2* and *SFB2*, we examined whether overexpression of *SMY2* suppresses the temperature-sensitive growth defect of the *sec24-20* mutant that is lacking *SFB2* (*sec24-20Δsfb2*). Chromosomal disruption of *SFB2* did not exacerbate the temperature-sensitive growth defect of the *sec24-20* cells (data not shown). Both the high-copy (*2μ*) and the low-copy (*CEN*) expression of *SMY2* suppressed the growth defect of *sec24-20Δsfb2* cells at the restrictive temperature of 33°C (Figure 1C), indicating that endogenous Sfb2p is not involved in the suppression by overexpression of *SMY2*. We therefore concluded that *SMY2* is a novel suppressor of the *sec24-20* mutant.

### Smy2p and its homologue Ypl105cp

**Figure 2:**
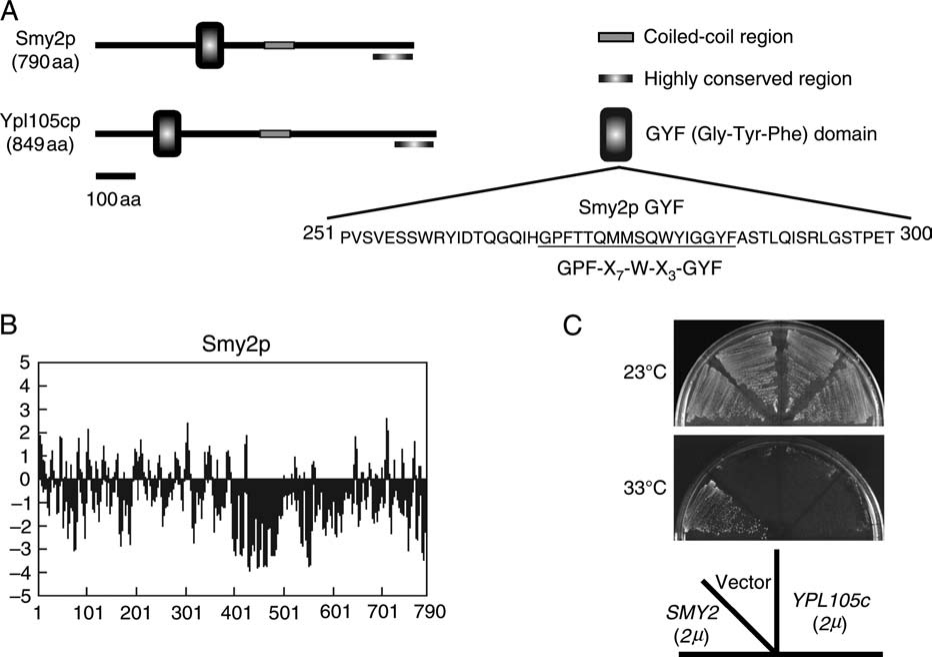
Smy2p and Ypl105cp. A) Schematic representation of Smy2p and its homologue Ypl105cp indicating conserved regions and consensus motifs present at comparable sites. Amino acid sequences around the GYF domain of Smy2p and consensus sequence of the domain are also indicated. B) Hydropathy profile of Smy2p calculated as described by Kyte and Doolittle (72). C) *sec24-20* (YNH2) cells containing pSMY1 (*SMY2*, *2μ*), pYPL1 (YPL105c, *2μ*) or pRS426 (vector) were streaked onto MVD plates and incubated at the indicated temperatures for 3 days. The amino acids underlined denote the consensus motif of the GYF domain.

*SMY2* encodes a 790 amino acid residue protein with a predicted molecular mass of 87 kDa. As shown in Figure 2A, Smy2p is predicted to contain a GYF domain, a proline-rich sequence binding module (46, 47), and a coiled-coil region by the smart program (48, 49). Its hydropathy profile indicates that Smy2p is a hydrophilic protein containing neither signal sequence nor transmembrane domain (Figure 2B). The *Saccharomyces* genome also contains an uncharacterized ORF YPL105c encoding an 849 amino acid residue protein with 30% overall identity to Smy2p (Figure 2A) (48–50). Besides the GYF and coiled-coil domains, the region corresponding to carboxy-terminal 100 amino acids is highly conserved between Smy2p and Ypl105cp. However, overexpression of YPL105c failed to suppress the temperature-sensitive growth defect of *sec24-20* cells (Figure 2C). To determine whether *SMY2* and YPL105c are required for growth, we constructed a diploid strain that one copy of each gene was disrupted. Tetrad analysis of the strain revealed that neither single nor double disruption of the genes affects growth or ER-to-Golgi transport (data not shown).

### Chromosomal disruption of *SMY2* exhibits a synthetic lethal interaction with the *sec24-20* mutation

**Figure 3:**
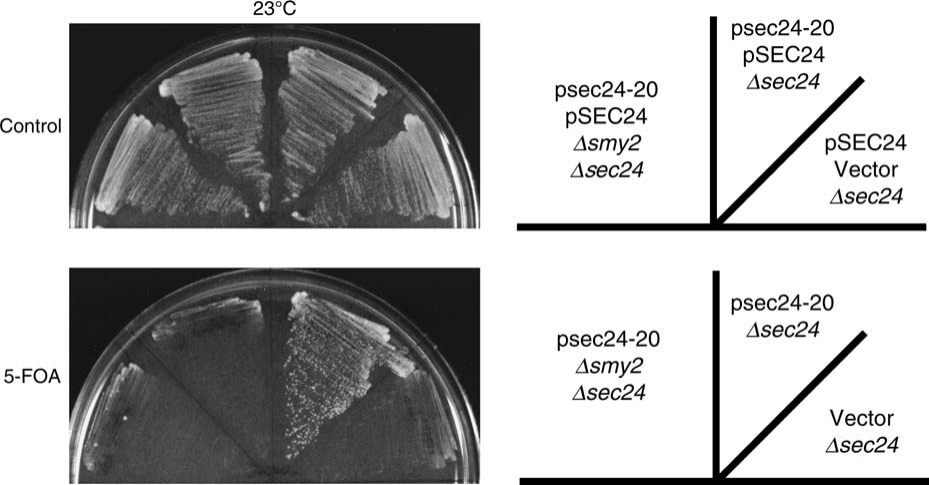
*SMY2* disruption exhibits severe growth defect in the *sec24-20* background *Δsec24* cells being rescued by pSEC24 (pAN1; *SEC24*, *CEN*, *URA3*) (YKH3) and *Δsec24Δsmy2* cells being rescued by pSEC24 (pAN1; *SEC24*, *CEN*, *URA3*) (YNH3) were transformed with psec24-20 (pAN12; *sec24-20*, *CEN*, *TRP1*) or pRS314 (vector). The transformants were streaked onto MVD (control) and MVD containing 5-FOA (5-FOA) plates and incubated at 23°C for 3 days.

To further examine the genetic interaction between *SEC24* and *SMY2*, we tested whether the chromosomal disruption of *SMY2* affects the growth phenotype of the *sec24-20* mutant. First, the chromosomal disruption of *SMY2* (*Δsmy2*) was introduced into a yeast strain YKH3, which contained the *sec24* null (*Δsec24*) mutation but was rescued by the plasmid pSEC24 (pAN1; *SEC24*, *CEN*, *URA3*), to generate a new yeast strain YNH3 (*Δsec24Δsmy2*). *Δsec24* (YKH3) and *Δsec24Δsmy2* (YNH3) strains were then transformed with the plasmid psec24-20 (pAN12; *sec24-20*, *CEN*, *TRP1*), and the resulting transformants were streaked onto selective media (MVD) plates containing 5-fluoroorotic acid (5-FOA), which allow only the cells lacking the functional *URA3* gene to grow. As shown in Figure 3, *Δsec24* (YKH3) cells containing psec24-20 (pAN12; *sec24-20*, *CEN*, *TRP1*) grew on 5-FOA plates, indicating that pSEC24 (pAN1; *SEC24*, *CEN*, *URA3*) was replaced by psec24-20 (pAN12; *sec24-20*, *CEN*, *TRP1*) to rescue the *sec24* null mutation. However, *Δsec24Δsmy2* (YNH3) cells containing psec24-20 (pAN12; *sec24-20*, *CEN*, *TRP1*) failed to grow on 5-FOA plates, indicating that the plasmid replacement was not achieved in the absence of *SMY2* and that the *sec24-20* mutation is synthetically lethal with the chromosomal disruption of *SMY2*.

### *SMY2* exhibits genetic interactions with several genes involved in the ER-to-Golgi transport

The strong genetic interaction with *SEC24* prompted us to examine whether *SMY2* also exhibits interactions with other genes involved in vesicular transport. First, the high-copy (*2μ*) *SMY2* plasmid was introduced into various temperature-sensitive mutants defective in vesicular transport between the ER and the Golgi, and the suppression activity was tested (Table 1). The high-copy (*2μ*) expression of *SMY2* suppressed the temperature-sensitive growth defect of the following mutants defective in ER-to-Golgi transport: *sec16-2* (COPII vesicle formation), *sec22-3* and *bet1-1* (ER–Golgi SNAREs), and *sec34-1* and *sec35-1* (vesicle tethering to the Golgi), but not mutants defective in vesicle fusion or Golgi-to-ER transport. The low-copy (*CEN*) expression of *SMY2* could suppress only the growth defect of *sec24-20*.

**Table 1:** Effect of *SMY2* overexpression on the growth of temperature-sensitive mutants defective in vesicular transport between the ER and the Golgi^a^

			Incubation temperature (°C)						
			
Function	Strain	Plasmid	26	28	30	32	34	35	37
COPII vesicle formation	*sec12-4*	vector	+	+	+	±	−	−	−
		*SMY2*(*2μ*)	+	+	+	+	−	−	−
	*sar1-2*	vector	+	+	+	+	+	+	−
		*SMY2*(*2μ*)	+	+	+	+	+	+	−
	*sec23-1*	vector	+	+	−	−	−	−	−
		*SMY2*(*2μ*)	+	+	±	−	−	−	−
	*sec23-2*	vector	+	+	+	−	−	−	−
		*SMY2*(*2μ*)	+	+	+	−	−	−	−
	*sec13-1*	vector	+	+	+	±	−	−	−
		*SMY2*(*2μ*)	+	+	+	+	−	−	−
	*sec24-20*	vector	+	+	±	−	−	−	−
		*SMY2*(*2μ*)	+	+	+	+	+	+	±
	*sec31-1*	vector	+	+	+	+	+	+	±
		*SMY2*(*2μ*)	+	+	+	+	+	+	+
	*sec16-2*	vector	+	+	−	−	−	−	−
		*SMY2*(*2μ*)	+	+	+	+	±	−	−
ER–Golgi SNAREs	*sec22-3*	vector	+	+	−	−	−	−	−
		*SMY2*(*2μ*)	+	+	+	+	+	±	−
	*bet1-1*	vector	+	+	+	+	−	−	−
		*SMY2*(*2μ*)	+	+	+	+	+	+	+
Vesicle tethering to the Golgi	*sec34-1*	vector	+	+	+	±	−	−	−
		*SMY2*(*2μ*)	+	+	+	+	+	+	+
	*sec35-1*	vector	+	+	+	−	−	−	−
		*SMY2*(*2μ*)	+	+	+	+	+	−	−
Vesicle fusion	*sec17-1*	vector	+	+	+	+	−	−	−
		*SMY2*(*2μ*)	+	+	+	+	±	−	−
	*sec18-1*	vector	+	±	−	−	−	−	−
		*SMY2*(*2μ*)	+	+	−	−	−	−	−
Golgi-to-ER transport	*sec20-1*	vector	+	+	±	−	−	−	−
		*SMY2*(*2μ*)	+	+	±	−	−	−	−
	*sec21-1*	vector	+	+	+	−	−	−	−
		*SMY2*(*2μ*)	+	+	+	−	−	−	−
	*sec27-1*	vector	+	+	±	−	−	−	−
		*SMY2*(*2μ*)	+	+	+	−	−	−	−
	*ret1-1*	vector	+	+	+	+	+	−	−
		*SMY2*(*2μ*)	+	+	+	+	±	−	−
	*ret3-1*	vector	+	+	+	+	±	−	−
		*SMY2*(*2μ*)	+	+	+	+	±	−	−

a Mutants containing pSMY1 (*SMY2*, *2μ*) or pRS426 (vector) were incubated on MVD plate for 3–4 days at the indicated temperatures, respectively.

We then examined whether the chromosomal disruption of *SMY2* affects the temperature-sensitive growth of mutants defective in ER-to-Golgi transport. Among the strains tested, we found that *Δsmy2* exhibited weak synthetic negative interactions with *sec13-1* (COPII vesicle formation) and *sec22-3* (ER–Golgi SNARE) (data not shown).

Together, our genetic observations suggest that *SMY2* is involved in the ER-to-Golgi transport, especially in the COPII vesicle formation.

### Smy2p localization

**Figure 4:**
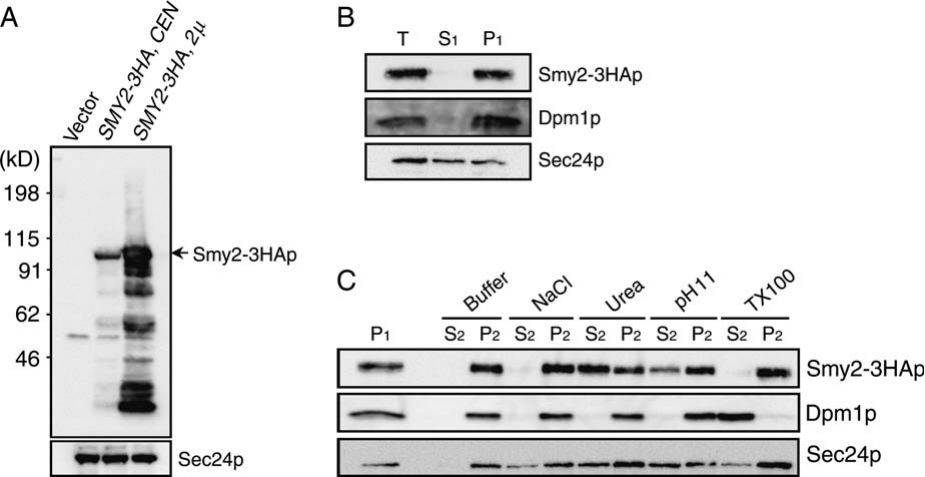
Smy2p is a peripheral membrane protein. A) Whole cell lysates (45 μg protein equivalents) from *Δsmy2* (YNH4) cells containing pSMY4 (*SMY2-3HA*, *CEN*), pSMY2 (*SMY2-3HA*, *2μ*) or pRS316 (vector) were resolved by SDS–PAGE and immunoblotted with the anti-HA or anti-Sec24p antibody. B) A whole cell lysate from *Δsmy2* (YNH4) cells containing pSMY4 (*SMY2-3HA*, *CEN*) was centrifuged at 100 000 × ***g***, and the resulting supernatant (S_1_) and pellet (P_1_) fractions were resolved by SDS–PAGE and immunoblotted with the anti-HA, anti-Sec24p or anti-Dpm1p antibody. C) Aliquots of the P_1_ fraction were treated with buffer, buffer containing 0.5 m NaCl (NaCl), 2.5 m urea (Urea), 0.1 m Na_2_CO_3_ (pH 11) or 1% Triton-X-100 (TX100). Samples were centrifuged at 100 000 × ***g***, and the resulting supernatant (S_2_) and pellet (P_2_) fractions were analyzed as in B.

To examine the intracellular distribution of Smy2p, three repeats of the influenza virus hemagglutinin (HA) epitope were tagged at its carboxyl terminus. Epitope-tagged *SMY2* (*SMY2-3HA*) was judged to be functional because it suppressed the *sec24-20* phenotypes to the same extent as untagged *SMY2* (data not shown). *SMY2-3HA* was introduced into *Δsmy2* (YNH4) strain by either a low-copy (*CEN*) or a high-copy (*2μ*) vector, and whole cell lysates prepared from the transformants were analyzed by immunoblotting with the anti-HA antibody (Figure 4A). Smy2-3HAp was detected as a protein with apparent molecular mass of 100 kDa that is higher than the predicted molecular mass of 87 kDa, probably because of its high isoelectric point of 8.97 as predicted by its amino acid composition. While the suppression activity for the *sec24-20* mutation was comparable (Figure 1A), the low-copy (*CEN*) *SMY2-3HA* plasmid gave much lower expression of Smy2-3HAp than the high-copy (*2μ*) plasmid.

Smy2p is predicted to be a hydrophilic protein containing neither signal sequence nor transmembrane domain (Figure 2B). To examine possible membrane association of Smy2p, the whole cell lysate from *Δsmy2* (YNH4) cells containing the low-copy (*CEN*) *SMY2-3HA* plasmid was subjected to 100 000 × ***g*** centrifugation, and the resulting supernatant (S_1_) and pellet (P_1_) fractions were analyzed by immunoblotting (Figure 4B). Unexpectedly, whereas the peripheral membrane protein Sec24p was detected in both S_1_ and P_1_ fractions, Smy2-3HAp was detected only in the P_1_ fraction like the integral membrane protein Dpm1p (51, 52). To further examine the nature of membrane association of Smy2p, the P_1_ fraction was treated with buffer containing 0.5 m NaCl (NaCl), 2.5 m urea (Urea), 0.1 m Na_2_CO_3_ (pH 11) or 1% Triton-X-100 (TX100), centrifuged at 100 000 × ***g*** to separate the supernatant (S_2_) and pellet (P_2_) fractions and then analyzed by immunoblotting. As shown in Figure 4C, the integral membrane protein Dpm1p was solubilized only by 1% Triton-X-100, but Smy2-3HAp was efficiently solubilized by 2.5 m urea or 0.1 m Na_2_CO_3_ as the peripheral membrane protein Sec24p. These results strongly suggest that Smy2p is a peripheral membrane protein with no soluble pool, but not an integral membrane protein.

**Figure 5:**
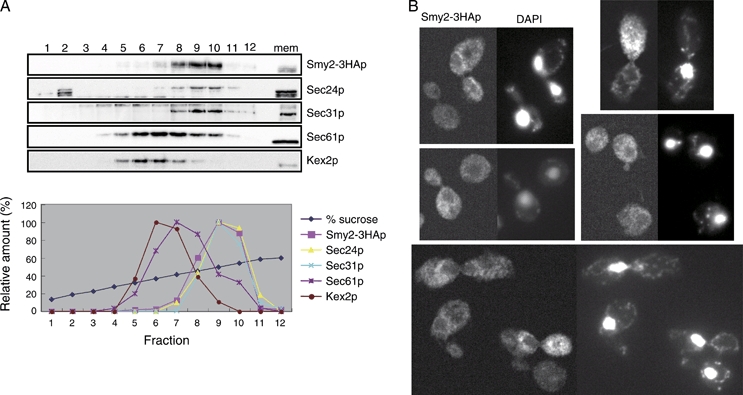
Smy2p is localized on ER membrane. A) A whole cell lysate from *Δsmy2* (YNH4) cells containing pSMY4 (*SMY2-3HA*, *CEN*) was centrifuged at 100 000 × ***g***. The resulting pellet fraction (mem) was resuspended in sucrose solution and separated on a 20–60% sucrose density gradient. Twelve fractions were collected from the top, resolved by SDS–PAGE and immunoblotted with the anti-HA, anti-Sec24p, anti-Sec31p, anti-Sec61p or anti-Kex2p antibody. Relative amounts of these proteins in each fraction were quantified by densitometry of immunoblots. B) Yeast cells used in A were examined by indirect immunofluorescence with the anti-HA antibody (left panels in the pair). DAPI staining was used to visualize the nuclei (right panels in the pair).

As our genetic observation suggests the involvement of Smy2p in COPII vesicle formation, the most plausible intracellular localization of Smy2p may be the ER membrane. To address this, the 100 000 × ***g*** pellet fraction of the lysate was subjected to a subcellular fractionation analysis by velocity sedimentation on a sucrose density gradient, and the distribution of Smy2-3HAp was compared with that of marker proteins of the ER and the Golgi by immunoblotting (Figure 5A). Notably, Smy2-3HAp was cosedimented with COPII components Sec24p and Sec31p in the fractions 8–10 in which the ER marker Sec61p was more abundant than the Golgi marker Kex2p. This result suggests that Smy2p is localized on the surface of the ER together with COPII components. Similar results were obtained from the subcellular fractionation analysis by flotation equilibrium in a sucrose density gradient, confirming that Smy2p associates with membranes but not with cytoskeletons (data not shown).

Finally, we examined the intracellular localization of Smy2p by microscopic analysis. *Δsmy2* (YNH4) cells containing the low-copy (*CEN*) *SMY2-3HA* plasmid were subjected to indirect immunofluorescence analysis with the anti-HA antibody. As shown in Figure 5B, many, if not all, cells exhibited concentrated staining around the 4,6-diamino-2-phenylindole (DAPI)-stained nucleus, reminiscent of the perinuclear localization of ER-resident proteins (53, 54). In addition, cells often exhibited weak punctate staining dispersed in the cell body. This staining pattern of Smy2-3HAp was similar to that of Sec16p, a peripheral membrane protein localized on the ER (26).

Together, these biochemical and morphological observations suggest that Smy2p is a peripheral membrane protein associated with the COPII-enriched ER membrane.

### Smy2p is not present on COPII vesicles formed *in vitro*

**Figure 6:**
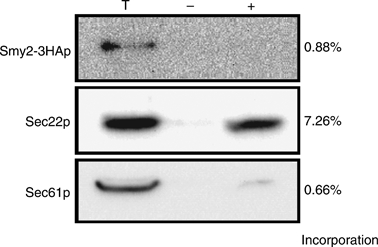
Smy2p is not incorporated into COPII vesicles. Salt-washed microsomes prepared from *Δsmy2* (YNH4) cells containing pSMY4 (*SMY2-3HA*, *CEN*) were incubated with (+) or without (−) purified COPII proteins in the presence of guanyl-5′-yl imidodiphosphate. One tenth of the total reaction (T) and the budded COPII vesicles separated from donor membranes by centrifugation were resolved by SDS–PAGE and immunoblotted with anti-HA, anti-Sec22p or anti-Sec61p antibody. Relative amounts of these proteins incorporated into COPII vesicles were quantified by densitometry of immunoblots.

Is Smy2p incorporated into COPII vesicles or statically localized on the ER? To address this, we performed an *in vitro* vesicle budding assay with purified COPII components and salt-washed microsomes, which were prepared from *Δsmy2* (YNH4) cells containing the low-copy (*CEN*) *SMY2-3HA* plasmid. The salt wash allowed Smy2-3HAp to remain associated with microsomes. As shown in Figure 6, the ER–Golgi SNARE Sec22p was efficiently incorporated into vesicle fraction, but Smy2-3HAp and the negative control Sec61p were not. Thus, Smy2p is likely to localize statically on the ER membrane.

We tried to compare the efficiency of vesicle formation among the salt-washed microsomes, which were prepared from YNH4 (*Δsmy2*) cells containing the low-copy (*CEN*) or the high-copy (*2μ*) *SMY2-3HA* plasmid or an empty vector. As far as we examined, however, no difference was observed under our experimental conditions that utilize the wild-type COPII components for vesicle formation (data not shown).

### Smy2p and a COPII subcomplex, Sec23p/Sec24p, are coimmunoprecipitated in specific COPII mutants

**Figure 7:**
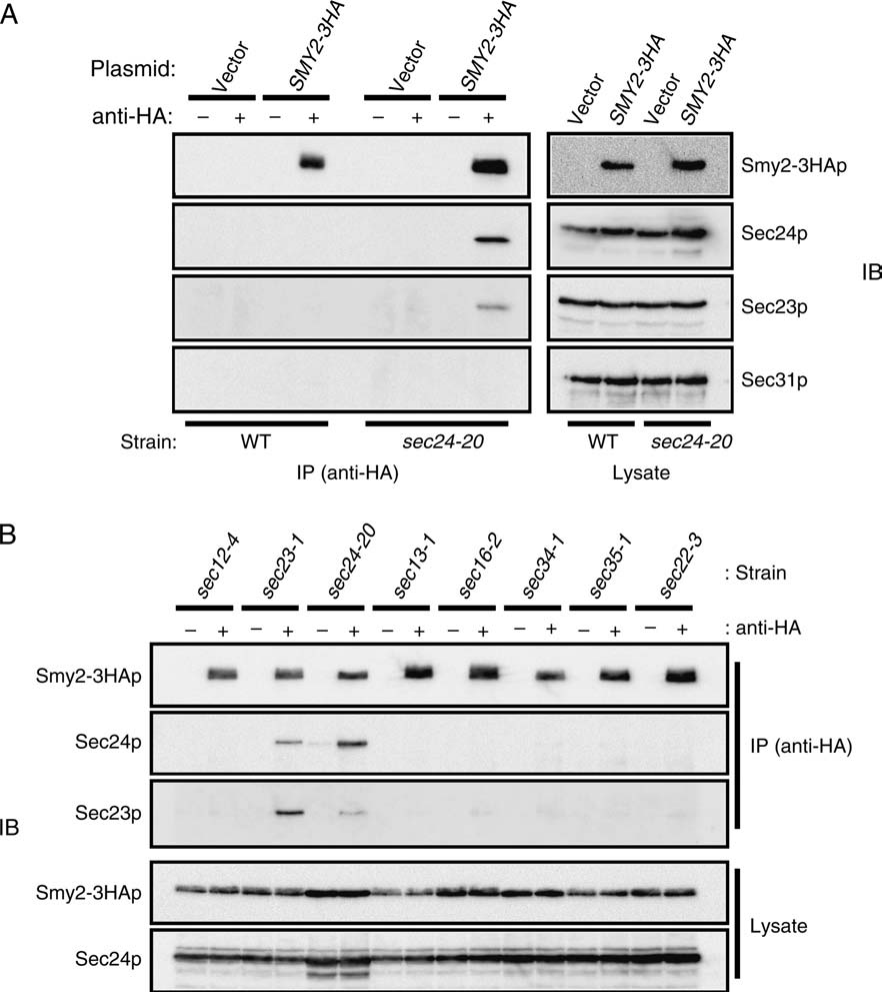
COPII subcomplex Sec23p/Sec24p is coprecipitated with Smy2p in *sec23* and *sec24* backgrounds. A) Whole cell lysates from wild-type (WT; YNH1) and *sec24-20* (YNH2) cells containing pSMY4 (*SMY2-3HA*, *CEN*) or pRS316 (Vector) (cultured at 23°C) were solubilized with 1% Triton-X-100. After centrifugation at 17 400 × ***g*** for 30 min at 4°C, supernatants were subjected to immunoprecipitation with (+) or without (−) the anti-HA antibody. The lysates (Lysate) and immunoprecipitates (IP) were resolved by SDS–PAGE and immunoblotted (IB) with the anti-HA, anti-Sec24p, anti-Sec23p or anti-Sec31p antibody. B) Whole cell lysates from *sec12-4* (MBY10-7A), *sec23-1* (RSY282), *sec24-20* (YNH2), *sec13-1* (RSY266), *sec16-2* (RSY268), *sec34-1* (YNH5), *sec35-1* (YNH6) and *sec22-3* (RSY942) cells containing pSMY4 (cultured at 23°C) were analyzed by immunoprecipitation as in A.

The results obtained so far support the idea that Smy2p might be functioning during COPII vesicle formation together with COPII components, especially with Sec24p. We therefore decided to examine whether Smy2p physically interacts with COPII components. In order to investigate their biochemical interaction in various mutant backgrounds, we performed coimmunoprecipitation experiments using Triton-soluble fractions of whole cell lysates, even though they contained small amounts of soluble Smy2-3HAp and COPII components (Figure 4). First, whole cell lysates from wild-type (YNH1) and *sec24-20* (YNH2) cells containing the low-copy (*CEN*) *SMY2-3HA* plasmid (cultured at 23°C) were solubilized by 1% Triton-X-100, centrifuged at 17 400 × ***g*** for 30 min at 4°C and the resulting supernatants were subjected to immunoprecipitation with the anti-HA antibody. Immunoprecipitates were then analyzed by immunoblotting with antibodies against COPII components. As shown in Figure 7A, we were able to pull down Smy2-3HAp with the anti-HA antibody from the Triton-soluble fractions of both strains and found that Sec24p and Sec23p, but not Sec31p, were coimmunoprecipitated with Smy2-3HAp in the *sec24-20* mutant background. In contrast, no coimmunoprecipitation was observed in the wild-type background. Next, to examine whether this protein–protein interaction is specific for the *sec24-20* background or is a general consequence of the reduced ER-to-Golgi transport, we performed the coimmunoprecipitation experiments using the Triton-soluble fractions of whole cell lysates prepared from the following temperature-sensitive mutants defective in ER-to-Golgi transport (cultured at 23°C): *sec12-4*, *sec13-1*, *sec16-2*, *sec23-1*, *sec24-20* (COPII vesicle formation), *sec34-1*, *sec35-1* (vesicle tethering to the Golgi) and *sec22-3* (ER–Golgi SNARE), which contain the low-copy (*CEN*) *SMY2-3HA* plasmid. Again, Smy2-3HAp was successfully recovered from the Triton-soluble fractions from all strains tested (Figure 7B). Surprisingly, the coimmunoprecipitation was observed in *sec23-1* and *sec24-20*, but not in other mutant backgrounds. Similar results were obtained from whole cell lysates from the mutants incubated at the restrictive temperature of 33°C at which the ER-to-Golgi transport is severely inhibited (data not shown). These results strongly suggest that the fraction of Smy2-3HAp solubilized by Triton-X-100 treatment was indeed interacting with the Sec23p/Sec24p subcomplex only in the *sec24-20* or *sec23-1* background, which seems to be a specific consequence of the dysfunction of Sec23p or Sec24p, rather than the general consequence of reduced ER-to-Golgi transport.

### Two-hybrid interaction between Smy2p and Sec23p

The interaction between Smy2p and the Sec23p/Sec24p subcomplex was also examined by the yeast two-hybrid system. Smy2p was fused to the *Lex*A-DNA binding domain and tested for interaction with Sec23p and Sec24p, which were connected to the B42 acidic activation domain. Interaction was detected by the level of β-galactosidase expression in yeast from a *Lac*Z reporter gene carrying *Lex*A operator sites in the promoter. Unexpectedly, interaction was detected between Smy2p and Sec23p, but not between Smy2p and Sec24p (Table 2). The β-galactosidase activity for this interaction was much lower than that for the highly stable interaction between Sec23p and Sec24p (as a positive control), suggesting that Smy2p weakly or transiently associates with Sec23p. Nevertheless, this two-hybrid interaction seems to represent, at least, a part of the nature of coimmunoprecipitation between Smy2p and the Sec23p/Sec24p subcomplex.

**Table 2:** Two-hybrid interaction between Smy2p and Sec23p^a^

		B42 acidic activation domain fused to		
		
		no fusion	SEC23	SEC24
*Lex*A-DNA binding domain fused to	no fusion	4.58 ± 0.54	1.09 ± 0.45	2.11 ± 1.32
	*SMY2*	3.57 ± 1.21	26.03 ± 11.15	1.62 ± 0.53
	*SEC24*	7.53 ± 1.77	401.40 ± 41.23	Not determined

a β-Galactosidase activity (units). EGY48 cells containing plasmids encoding a *Lex*A fusion protein (pEG202, pEG-SMY2 or pEG-SEC24), a B42 fusion protein (pJG4-5, pJG-SEC23 or pJG-SEC24) and a reporter plasmid (pSH18-34) were grown in raffinose/galactose medium for 10 h before the assay to induce expression of B42 fusion proteins.

### Overexpression of Smy2p suppresses the *sec24-20* phenotypes through the interaction with Sec23p/Sec24p subcomplex

**Figure 8:**
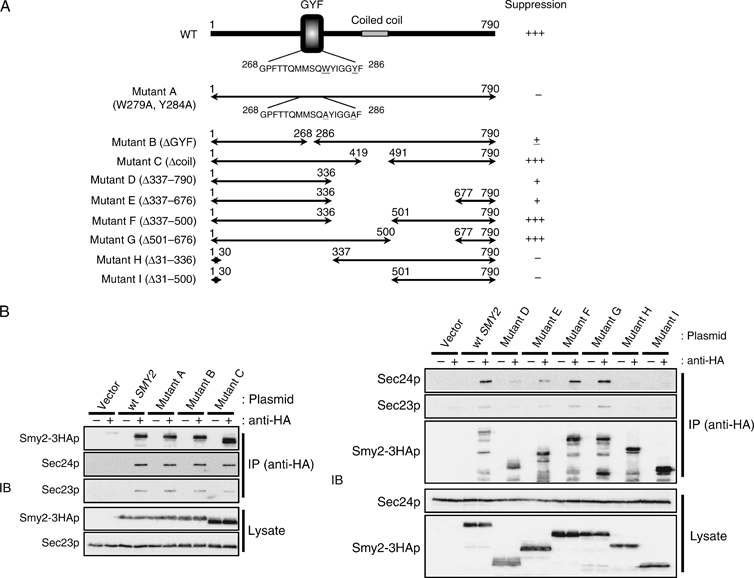
Mutational analysis of Smy2p. A) Schematic representation of mutant Smy2p. The amino acid numbers corresponding to boundaries of each fragment are indicated. Each *smy2* construct was introduced into *sec24-20* (YNH2) cells with a low-copy vector pRS316 (*URA3*, *CEN*), and its suppression activity was examined as in Figure 1A. After three independent experiments, the relative strength of suppression was indicated for each construct. B) Whole cell lysates from the transformants used in A (cultured at 23°C) were analyzed by immunoprecipitation as in Figure 7A. The underlined indicate the original amino acid residues in the WT GYF domain and the corresponding mutated amino acid residues in Mutant A.

We performed a mutational analysis of Smy2p to identify the regions required for the suppression of *sec24-20* phenotypes and for the interaction with Sec23p/Sec24p subcomplex. First, a series of mutant *smy2* genes with 3HA epitope at their carboxyl termini were constructed and introduced on the low-copy (*CEN*) vector into *sec24-20* (YNH2) cells to examine their suppression activity (Figure 8A). The suppression activity was severely reduced when the consensus sequence of the GYF domain (GPF-X_7_-W-X_3_-GYF) was disrupted by amino acid substitutions (mutant A) or deletion (mutant B). The deletion of the coiled-coil domain did not affect the suppression activity (mutant C). We also found that the amino-terminal region alone, which contained the GYF domain (amino acid residues 1–336), exhibited a low level of suppression activity (mutant D), and this was restored to the wild-type level by fusion with the carboxyl-terminal portion containing amino acid residues 501–676 (mutant F) but not with residues 677–790 (mutant E). Another amino- and carboxyl-terminal fusion containing amino acid residues 1–500 and 677–790 also showed a high suppression activity (mutant G). However, the carboxyl-terminal portion alone was insufficient for the suppression (mutants H and I). These results suggest that the combination of the intact GYF domain and a certain length of the carboxyl-terminal domain is required for the suppression.

We then examined whether these mutant Smy2p interact with Sec23p/Sec24p subcomplex by coimmunoprecipitation analysis in the *sec24-20* background as described above. As shown in Figure 8B, the Sec23p/Sec24p subcomplex was coimmunoprecipitated with the GYF mutants (mutants A and B) or the coiled-coil mutant (mutant C), suggesting that these domains are not involved in the interaction. Notably, the amino-terminal portion alone (amino acid residues 1–336) failed to coimmunoprecipitate Sec23p/Sec24p (mutant D), and the fusion with a certain length of the carboxyl-terminal portion restored the coimmunoprecipitation (mutants F and G). Again, the carboxyl-terminal portion alone exhibited no coimmunoprecipitation (mutants H and I). These results suggest that neither the GYF nor the coiled-coil domain alone, but a larger part of Smy2p, is required for the interaction with Sec23p/Sec24p subcomplex.

Taken together, these genetic and biochemical analyses indicate that all mutant Smy2p with the suppression activity could coimmunoprecipitate Sec23p/Sec24p and that the strength of the suppression well correlated with the amount of the Sec23p/Sec24p subcomplex coimmunoprecipitated. Thus, the interaction between Smy2p and the Sec23p/Sec24p subcomplex is probably a prerequisite for the suppression of the *sec24-20* phenotypes by overexpression of *SMY2*.

## Discussion

In this study, we isolated *SMY2* as a suppressor of the temperature-sensitive *sec24-20* mutant. We first excluded the possibility that endogenous Sfb2p (Iss1p), a functionally redundant *SEC24* homologue, is involved in the suppression by overexpression of *SMY2* (Figure 1C). It is more unlikely that the suppression involves endogenous Sfb3p (Lst1p) as its overexpression cannot suppress the temperature-sensitive growth defect of the *sec24* mutants (17, 18). Thus, we concluded that *SMY2* is a novel suppressor of the *sec24-20* mutant and further characterized its properties.

The *Saccharomyces* genome contains the *SMY2* homologue YPL105c (48–50), but its overexpression failed to suppress any mutants defective in vesicular transport between the ER and the Golgi (Figure 2C and our unpublished observation). In addition, immunofluorescence staining of Ypl105c-3HAp did not show the perinuclear ER-like pattern, as was the case with Smy2-3HAp (our unpublished observation). Thus, we consider that despite the sequence similarity, Ypl105cp is not the functional Smy2p homologue.

Genetic analysis revealed that all mutations suppressed by the high-copy (*2μ*) expression of *SMY2* are linked to the ER-to-Golgi transport (Table 1). *SMY2* exhibited particularly strong genetic interaction with *SEC24* (Figures 1 and 3). This may suggest that Smy2p functions co-operatively with Sec24p in COPII vesicle formation. However, considering other mutations suppressed by the high-copy (*2μ*) expression of *SMY2*, other possibilities cannot be ruled out. For example, Smy2p might function in tethering (*sec34-1* and *sec35-1*) and/or fusion (*sec22-3* and *bet1-1*) of ER-derived vesicle to the Golgi membrane. The intimate relationship between COPII vesicle formation and vesicle tethering/fusion to the Golgi membrane in mammalian cells (55) also leaves a possibility that Smy2p might affect indirectly on these aspects of ER-to-Golgi transport from the stage of COPII vesicle formation in yeast.

Biochemical and morphological analyses suggested that Smy2p is a peripheral ER membrane protein cofractionated with COPII components (Figures 4 and 5). However, *in vitro* vesicle budding assay revealed that Smy2p is not incorporated into COPII vesicles (Figure 6), suggesting that Smy2p acts as neither coat nor cargo protein, but is statically localized on the ER and facilitates COPII vesicle formation.

Coimmunoprecipitation analysis revealed that Smy2p specifically interacts with the Sec23p/Sec24p subcomplex in *sec24-20* and *sec23-1* mutants (Figure 7). This observation may represent prolonged interaction of the proteins in these mutant backgrounds and implies that Smy2p assists some Sec23p/Sec24p-deficient process during COPII vesicle formation, while the interaction may be too transient to be detected by coimmunoprecipitation in wild-type cells. We further found that the interaction between Smy2p and the Sec23p/Sec24p subcomplex appears to be a prerequisite for the suppression of the *sec24-20* phenotypes by overexpression of *SMY2* (Figure 8). What is then the mechanism of the suppression? The *sec24-20* gene has a mutation by which the 897th codon (TGG) encoding tryptophan (W897) is changed to a termination codon (TGA) (our unpublished observation), resulting in producing Sec24-20p, a truncated Sec24p lacking the carboxyl-terminal 30 amino acids. This short carboxyl-terminal region is shown to locate on the membrane-proximal surface (56). Moreover, W897 is located in the cargo recognition ‘A-site’ and is shown to be important for Sed5p binding (13, 14, 57). As the *sec24-20* mutant exhibits growth arrest and the blockage of ER-to-Golgi transport at the restrictive temperature, without obvious accumulation of vesicles (17), we consider that the Sec23p/Sec24-20p subcomplex probably becomes defective in COPII vesicle formation at high temperatures because of the weakened interaction with membranes and/or membrane proteins such as Sed5p. If this is the case, the suppression by excess Smy2p could be accounted for by the possibility that Smy2p acts as a scaffold or a platform for the assembly of COPII coat, which directly binds Sec23p/Sec24p, like the essential scaffold protein Sec16p (26–29). An increased amount of Smy2p may provide the additional scaffold or platform, leading to the compensation for the impaired function of Sec23p/Sec24-20p. This idea is supported by the suppression of the *sec16-2* mutation by the high-copy (*2μ*) expression of *SMY2* (Table 1) and the two-hybrid interaction between Smy2p and Sec23p (Table 2). Synthetic lethal interaction between *sec24-20* and chromosomal deletion of *SMY2* (Figure 3) is also consistent with this idea. Finally, it is unlikely that Smy2p modulates the GTPase cycle of Sar1p because the overexpression of *SMY2* did not suppress or exacerbate the temperature-sensitive growth defect of the mutants, *sec23-1*, *sec23-2* (encoding a GAP for Sar1p), *sec12-4* (encoding a GEF for Sar1p) and *sar1-2* (Table 1). Further extensive biochemical studies will be necessary for understanding the nature of defect in the *sec24-20* mutant and the mode of its suppression by Smy2p.

Mutational analysis of Smy2p revealed that its GYF domain, a proline-rich sequence binding module, is required for the suppression of the *sec24-20* phenotypes but not responsible for the interaction with Sec23p/Sec24p subcomplex (Figure 8). This suggests that the GYF domain mediates the interaction between Smy2p and another protein that is also required for the suppression of the *sec24-20* phenotypes. Genome-wide two-hybrid analysis has shown that Smy2p interacts with Msl5p and Mud2p, nuclear proteins involved in messenger RNA splicing (58). However, it is unlikely that these nuclear proteins facilitate the COPII vesicle formation co-operatively with Smy2p on the ER membrane. Identification of additional Smy2p-interacting protein will be helpful to understand the molecular mechanism by which Smy2p facilitates COPII vesicle formation.

Finally, our work strongly suggests that Smy2p is an accessory protein that facilitates COPII vesicle formation. Recently, such putative accessory proteins have also been identified on ER exit sites of mammalian cells. The nucleoside diphosphate kinase Nm23H2 facilitates COPII vesicle formation independent of its kinase activity (59). The protein kinase PCTAIRE, which has been identified as a Sec23p-interacting protein, requires its kinase activity for ER exit of secretory cargo but not for the interaction with Sec23p (60). In addition, the Ca^2+^-binding protein ALG-2 (apoptosis-linked gene 2) interacts with Sec31p in a Ca^2+^-dependent manner (61), and the phospholipase A1-related protein p125 interacts with Sec23p (62). These proteins are possibly required for modulating COPII vesicle formation and/or organizing ER exit sites. Besides Sec16p, Sed4p and the Yip1p–Yif1p–Yos1p complex (see *Introduction*), these findings together with our findings further support the view that, in living cells, COPII vesicle formation is a more complicated process than that reconstituted *in vitro* with minimal components. Identification and detailed characterization of accessory proteins may provide novel insights into the regulation of COPII vesicle formation under physiological conditions.

## Materials and Methods

### Yeast strains and media

*Saccharomyces cerevisiae* strains used in this study are listed in Table 3. Cells were grown in MVD medium [0.67% yeast nitrogen base without amino acids (Difco Laboratories Inc.) and 2% glucose], MCD medium [MVD containing 0.5% casamino acids (Difco Laboratories Inc.)] or SC-raffinose (0.67% yeast nitrogen base without amino acids, 0.06% dropout mix (63) and 2% raffinose) with appropriate supplementations.

**Table 3:** Yeast strains used in this study

Strain	Genotype	Reference/Source
YPH500	*MAT*α*ura3 trp1 his3 leu2 lys2 ade2*	(65)
YPH501	*MAT*a*/MAT*α*ura3/ura3 trp1/trp1 his3/his3 leu2/leu2 lys2/lys2 ade2/ade2*	(65)
YKH3	*MAT*a *ura3 trp1 his3 leu2 Δsec24::LEU2* containing pAN1 (*SEC24, URA3, CEN*)	(17)
YNH1	*MAT*a *ura3 trp1 his3 leu2 lys2 ade2 Δsec24::HIS3* containing pAN11 (*SEC24, TRP1, CEN*)	This study
YNH2	*MAT*a *ura3 trp1 his3 leu2 lys2 ade2 Δsec24::HIS3* containing pAN12 (*sec24-20, TRP1, CEN*)	This study
YNH3	*MAT*a *ura3 trp1 his3 leu2 Δsec24::LEU2 Δsmy2::HIS3* containing pAN1 (*SEC24, URA3, CEN*)	This study
YNH4	*MAT*a *ura3 trp1 his3 leu2 lys2 ade2 Δsmy2::HIS3*	This study
YNH5	*MAT*a *ura3 his3 lys2 sec34-1*	This study
YNH6	*MAT*a *ura3 trp1 leu2 lys2 sec35-1*	This study
YNH7	*MAT*a *ura3 trp1 his3 leu2 Δsec24::LEU2* containing pAN12 (*sec24-20, TRP1, CEN*)	This study
YNH8	*MAT*a *ura3 trp1 his3 leu2 Δsec24::LEU2 Δsfb2::HIS3* containing pAN12 (*sec24-20, TRP1, CEN*)	This study
MBY10-7A	*MAT*a *ura3 trp1 his3 his4 leu2 suc2 gal2 sec12-4*	(4)
TOY224	*MAT*α*ura3 trp1 his3 leu2 lys2 ade2 Δsar1::HIS3 Δpep4::ADE2* containing pMYY3-9 (*sar1*E112K (*sar1-2*)*, TRP1, CEN*)	(73)
RSY266	*MAT*a *ura3 his4 sec13-1*	R. Schekman^a^
RSY268	*MAT*a *ura3 sec16-2*	R. Schekman
RSY270	*MAT*a *ura3 his4 sec17-1*	R. Schekman
RSY11	*MAT*α*ura3 leu2 suc2 sec18-1*	R. Schekman
RSY276	*MAT*a *ura3 his4 sec20-1*	R. Schekman
RSY278	*MAT*a *ura3 his4 sec21-1*	R. Schekman
RSY942	*MAT*a *ura3 lys2 sec22-3*	R. Schekman
RSY282	*MAT*a *ura3 leu2 sec23-1*	R. Schekman
RSY639	*MAT*a *ura3 leu2 sec23-2*	R. Schekman
RSY1312	*MAT*a *ura3 trp1 leu2 sec27-1*	R. Schekman
RSY1004	*MAT*α*ura3 leu2 sec31-1*	R. Schekman
RSY958	*MAT*a *lys2 sec34-1*	R. Schekman
RSY962	*MAT*a *lys2 sec35-1*	R. Schekman
RSY944	*MAT*a *ura3 lys2 bet1-1*	R. Schekman
EGY101	*MAT*a *ura3 trp1 his3 leu2 suc2 ret1-1*	F. Letourneur^b^
FLY89	*MAT*a *ura3 trp1 his3 leu2 suc2 ret3-1*	F. Letourneur
EGY48	*MAT*α*ura3 trp1 his3 leu2::*LexAop-*LEU2*	OriGene Technologies Inc.

a University of California, Berkeley, Berkeley, CA, USA.

b UMR5086 CNRS/Universite Lyon I, Lyon, France.

### Plasmid construction

Construction of *SEC24* plasmids, pAN1, pAN11 and pAN12, was described previously (17). The DNA fragment containing *SMY2* was obtained from genomic DNA by polymerase chain reaction (PCR) with the primers containing a *Sac*I or *Not*I restriction site at their 5′ termini (5′-AGAGAGAGGCGGCCGCATACATCTGTCACGTAAACCATTG-3′ and 5′-AGAGAGAGGAGCTCGTGCCTACTGTGTGCAAAGATATG-3′), digested by *Sac*I and *Not*I and subcloned into the *Sac*I–*Not*I sites of pRS426 (64) and pRS316 (65) to produce pSMY1 and pSMY3, respectively. Similarly, the DNA fragment containing YPL105c was obtained by PCR with the primers containing a *Bam*HI or *Eco*RI site at their 5′ termini (5′-AGAGAGAGGGATCCGTTTGATGGTCGAGGTGTCCTTCC-3′ and 5′-AGAGAGAGGAATTCCACTTCTCATCTCCATTAAGAACC-3′), digested by *Bam*HI and *Eco*RI and subcloned into the *Bam*HI–*Eco*RI sites of pRS426 to produce pYPL1. The *SMY2* gene with three repeated HA (3HA) epitopes at the carboxyl terminus was generated as follows with pRS304-3HAc (a generous gift from K. Kohno), a plasmid containing the 3HA epitopes with a stop codon in the *Pst*I–*Eco*RI sites of pRS304 (65). The DNA fragment corresponding to the downstream 500 bp of *SMY2* was obtained by PCR with the primers containing *Eco*RI and *Xho*I sites at their 5′ termini (5′-AGAGAGAGGAATTCTGAAAGGAAAAGCTTCATAATTT-3′ and 5′-AGAGAGAGCTCGAGTTCACGAGAAGGTGTTGAAGGCCG-3′), digested with *Eco*RI and *Xho*I and subcloned into the *Eco*RI–*Xho*I sites of pRS304-3HAc to produce pRS304-3HAc-UTR. Then, the ORF of *SMY2* with its upstream 250 bp was obtained by PCR with the primers containing *Sac*I or *Pst*I sites at their 5′ termini (5′-AGAGAGAGGAGCTCATACATCTGTCACGTAAACCATTG-3′ and 5′-AGAGAGAGCTGCAGCGTGTTTTCTACCCTTCTTCTTGCT-3′), digested with *Sac*I and *Pst*I, and subcloned into the *Sac*I–*Pst*I sites of pRS304-3HAc-UTR to produce pRS304-SMY2-HAc-UTR. Finally, the DNA fragment containing *SMY2-3HA* construct was obtained by the *Pvu*II digestion of pRS304-SMY2-HAc-UTR and subcloned into the *Pvu*II site of pRS426 and pRS316 to produce pSMY2 and pSMY4, respectively. To create the mutant *smy2* genes encoding the proteins schematically represented in Figure 8A, the ORF of *SMY2* with its upstream 250 bp was subjected to PCR-mediated site-directed mutagenesis with the primers corresponding to each mutation. As described above, the resulting mutated ORFs were subcloned into pRS304-HAc-UTR, and then the fragments containing mutant *smy2-3HA* construct were subcloned into pRS316 to produce pSMY5 (mutant A) to pSMY13 (mutant I). pJG-SEC23, pJG-SEC24, pEG-SEC24 and pEG-SMY2 are the plasmids for yeast two-hybrid assay. Briefly, pJG-SEC23 and pJG-SEC24 are the plasmids that the ORFs of *SEC23* and *SEC24* with their downstream 350 bp were fused in frame with the B42 acidic activation domain at the *Xho*I site in pJG4-5 (OriGene Technologies Inc.), respectively. pEG-SEC24 is the plasmid that the ORF of *SEC24* with its downstream 350 bp was fused in frame with the *Lex*A-DNA binding domain at the *Not*I site in pEG202 (OriGene Technologies Inc.). Similarly, pEG-SMY2 was constructed with pEG202 and the ORF of *SMY2* with its downstream 550 bp.

### Antibodies

The anti-Sec22p, anti-Sec23p, anti-Sec24p, anti-Sec31p and anti-Sec61p antibodies were gifts from R. Schekman (University of California, Berkeley, Berkeley, CA, USA), and the anti-Gas1p antibody was from H. Riezman (University of Basel, Basel, Switzerland). The anti-CPY and anti-Kex2p antibodies were described previously (66, 67). Mouse monoclonal antibodies, anti-HA, 16B12, 12CA5 and anti-Dpm1p, were purchased from Berkeley Antibody, Roche Diagnostics and Molecular Probes.

### Metabolic labeling and immunoprecipitation of CPY and Gas1p

Pulse–chase experiments were performed as described previously (68, 69). Yeast cells grown exponentially in MCD medium were labeled with 25 μCi of Redivue PRO-MIX [^35^S] cell labeling mix (Amersham Pharmacia Biotech UK Ltd.) per 1 × 10^7^ cells and chased for appropriate times. Marker proteins, CPY and Gas1p were recovered from the same cell lysates by immunoprecipitation with anti-CPY and anti-Gas1p at 1:500 dilution, resolved by SDS–PAGE and visualized with a BAS2500 image analyzer (Fuji Photo Film). Amounts of mature forms of CPY and Gas1p were quantified with Image Gauge version 3.45 software (Fuji Photo Film) and expressed as %maturation.

### Immunofluorescence microscopy

Indirect immunofluorescence was observed as described previously (17) with the anti-HA (16B12) antibody as the primary antibody and fluorescein 5(6)-isothiocyanate-conjugated goat anti-mouse IgG (Cappel Research Products, ICN, Inc.) as the secondary antibody. Nuclei were visualized by DAPI staining. Preparations were observed with a fluorescence microscope BX-60 (Olympus) equipped with a confocal laser scanner unit CSU10 (Yokogawa Electric Corp.). Images were captured by a high-resolution digital charge-coupled device camera ORCA-ER (Hamamatsu Photonics) and processed with IPLab software (Scanalytics Inc.).

### Subcellular fractionation

Subcellular fractionation was performed as described previously (70) with the following modification. A whole cell lysate prepared from log-phase yeast cells was centrifuged at 100 000 × ***g*** (model 100AT4 rotor; Hitachi Koki Co., Ltd.) for 20 min at 4°C. The resulting pellet fraction was resuspended in sucrose solution [10 mm HEPES–KOH, pH 7.4, 1 mm ethylenediaminetetraacetic acid (EDTA) and 12.5% sucrose] containing protease inhibitors (2 mm phenylmethylsulfonyl fluoride and 5 μg/mL each of chymostatin, leupeptin, antipain, pepstatin A and aprotinin), placed on a 20–60% sucrose density gradient and centrifuged at 217 000 ×***g*** (at the bottom of the tube) (model RPS-40T rotor; Hitachi Koki Co., Ltd.) for 2.5 h at 4°C. Twelve fractions were collected from the top and analyzed by SDS–PAGE and immunoblotting. To characterize membrane association of Smy2p, a yeast cell lysate was prepared and treated as follows. Log-phase yeast cells were spheroplasted and disrupted by agitation with glass beads in buffer 88 (20 mm HEPES–KOH, pH 6.8, 250 mm sorbitol, 150 mm KOAc and 5 mm MgOAc) containing protease inhibitors. Unbroken cells and debris were removed by centrifugation at 500 × ***g***, and aliquots of the cleared whole cell lysate were centrifuged at 100 000 × ***g*** (model 100AT4 rotor; Hitachi Koki Co., Ltd.) for 20 min at 4°C. The pellet fractions were resuspended in buffer 88, or buffer 88 containing 0.5 m NaCl, 2.5 m urea, 0.1 m Na_2_CO_3_ or 1% Triton-X-100, and incubated on ice for 30 min. Samples were then centrifuged at 100 000 × ***g*** (model 100AT3 rotor; Hitachi Koki Co., Ltd.) for 20 min at 4°C, and the resulting pellet and supernatant fractions were analyzed by SDS–PAGE and immunoblotting.

### *In vitro* vesicle budding

Microsome preparation and *in vitro* vesicle budding reactions were performed as described previously (70, 71). The vesicle fraction was analyzed by SDS–PAGE and immunoblotting.

### Coimmunoprecipitation

For coimmunoprecipitation, log-phase yeast cells were spheroplasted and disrupted by agitation with glass beads in lysis buffer (50 mm Tris–HCl, pH 7.5, 150 mm NaCl, 5 mm EDTA and 5% glycerol) containing protease inhibitors. After removing unbroken cells and debris, the whole cell lysate was solubilized with 1% Triton-X-100 in immunoprecipitate (IP) buffer (50 mm Tris–HCl, pH 7.5, 150 mm NaCl, 5 mm EDTA and 5% skim milk) containing protease inhibitors. After centrifugation at 17 400 × ***g*** for 30 min at 4°C, the resulting supernatant was rotated overnight with the protein G-immobilized anti-HA (12CA5) antibody at 4°C. The beads were washed four times with wash buffer (IP buffer without skim milk) and boiled in the SDS–PAGE sample buffer (50 mm Tris–HCl, pH 6.8, 1% sodium dodecyl sulfate, 10% glycerol and 0.01% bromophenol blue) containing 0.1 m dithiothreitol. The eluates were analyzed by SDS–PAGE and immunoblotting.

### Yeast two-hybrid assay

Sec23p and Sec24p were tested for binding to Smy2p with a DupLEX-A yeast two-hybrid system (OriGene Technologies Inc.). EGY48 strains were transformed with combinations of control or fusion protein plasmids described under *Plasmid construction* together with a *Lac*Z reporter plasmid pSH18-34. The resulting transformants were grown to a log phase in SC-raffinose medium, then galactose was added to a final concentration of 2% and growth continued for 10 h. β-Galactosidase assay with permeabilized cells was performed as described previously (63).
